# 

**Published:** 1895-11-02

**Authors:** 


### CONTENTS.

**Some Unutilized Resources of Civilisation** - - - - 73

**An Appeal to Appealers** - - - - - - 74

**Fifteen Thousand Pounds for the Charities** - - - - 74

**Administration of Anæsthetics** - - - - - - - 75

**The Fate of Colour-blind Sailors** - - - - - - - 76

**Annotations.**—Abnormality or Madness?—Guy's, and Mr. Gladstone's Appeal - - - - - - - - - - 77

**Medical Progress and Hospital Clinics—**

The Treatment of Abscess in Pott's Disease - - - - 79

Progress in Medicine.—Graves' Disease (*continued*) - - - 80

Progress in Rhinology - - - - - - - - - 83

New Appliances and Things Medical - - - - - - 84

**Notes and News** - - - - - - - - - - - - - 78

**The Institutional Workshop—**

Hospital Expenditure. — The Commissariat. IV. — The Nursing Staff in Proportion to Beds - - - - - - - 85

Metropolitan Hospital Sunday Fund - - - - - - - 86

Practical Departments.—Disinfection and the Existing Data for its Practice.—III. - - - - - - - - - 87

Hospital Festivals Meetings, &c. - - - - - - 87

Around the Hospitas - - - - - - - - - 88

Construction Notes - - - - - - - - - 88

**Scraps and Gleanings** - - - - - - - - - - 88

**The Book World of Medicine and Science** - - - - 89

### EXTRA SUPPLEMENT.—THE NURSING MIRROR.

**News from the Nursing World.**—The Royal National Pension Fund—The Prince of Wales at Newmarket Union—Princess Louise at Edinburgh—Our Needlework Competition—Paddington Green Children's Hospital—A Tragic End—The Hospital for Women at Birmingham—Three Years' Training—Ratepayers' Victory at Coventry—National Union of WomenWorkers—TheAlton Nurse Society—RugbyDistrict Nursing Association—St. John Ambulance at Leicester—Nuns as Nurses—Nurses for Johannesburg—Short Items - xxxiii
**Minor Appointments** - - - - - - - - - - xxxv
**Wants and Workers** - - - - - - - - - - xxxv
**Elementary Physiology for Nurses.** By C. F. Marshall, late Surgical Registrar, Hospital for Sick Children, Great Ormond Street. XIII.—The Nervous System (*continued*) - - - - - - - - - - - - - - xxxv
**Twenty-three Thousand Pounds for the Pension Fund** - - - - - - - - - - - - - - - xxxvi
**Where to Go** - - - - - - - - - - - - - - xxxvi
**Appointments** - - - - - - - - - - - - - xxxvi
**Novelties for Nurses** - - - - - - - - - - xxxvii
**Everybody's Opinion** - - - - - - - - - - xxxvii
**For Reading to the Sick** - - - - - - - - - xxxviii

